# A prediction model based on machine learning: prognosis of HBV-induced HCC male patients with smoking and drinking habits after local ablation treatment

**DOI:** 10.3389/fimmu.2025.1464863

**Published:** 2025-03-28

**Authors:** Han Shi, Caixia Hu

**Affiliations:** ^1^ Beijing You’an Hospital, Capital Medical University, Beijing, China; ^2^ Interventional therapy center for oncology, Beijing You ‘an Hospital, Capital Medical University, Beijin, China

**Keywords:** hepatocellular carcinoma, male, smoking and drinking, machine learning, nomogram

## Abstract

**Background:**

Liver cancer, particularly hepatocellular carcinoma (HCC), is a major health concern globally and in China, possibly shows recurrence after ablation treatment in high-risk patients. This study investigates the prognosis of early-stage male HCC patients with chronic hepatitis virus B (HBV) infection who also have long-term smoking and drinking habits, following local ablation treatment.

**Methods:**

Data from 257 patients treated at Capital Medical University, Beijing Youan Hospital from 2014 to 2022 were retrospectively analyzed. We first screened the variables by Lasso regression and random survival forest (RSF), followed by multivariate Cox regression analysis. Based on the screened variables after these steps, we performed and validated a nomogram to predict the survival status of these patients.

**Results:**

Our results indicated that monocytes and globulin are risk factors while pre-albumin (PALB) is protective after selected by Lasso, RSF and multivariate Cox regression, providing a robust tool for predicting overall survival and guiding treatment for high-risk HCC patients. With promising discrimination, accuracy and clinical applicability, our model was translated into a nomogram for practical use.

**Conclusion:**

Our prognostic model effectively identifies key risk factors such as monocytes, globulin and PALB, providing accurate predictions for HBV-induced male patients with smoking and drinking habits.

## Introduction

Liver cancer is one of the most severe malignant endpoint events resulting from various liver diseases. It remains the third leading cause of cancer-related death worldwide and is among the top five causes of cancer diagnosis and death in China. The most common form of liver cancer is hepatocellular carcinoma (HCC), which frequently occurs in patients with cirrhosis (1, 2).

The main treatments for HCC include surgery, ablation, intra-vascular treatment, radiotherapy, and chemotherapy. The choice of treatment varies depending on the stage of HCC. The Barcelona Clinic Liver Cancer (BCLC) staging system, which divides HCC into five stages (0 to D), is the most commonly used criteria for evaluating the progression of HCC. Early-stage HCC patients (BCLC 0 and A) respond well to local ablation treatment (3).However, despite the effectiveness of this treatment, there is still a high rate of intrahepatic metastases and vascular invasion post-ablation, leading to HCC recurrence (4).

Various risk factors contribute to the incidence of HCC, with viral infections being the most significant. Hepatitis B virus (HBV) infection is the primary cause of HCC in China. In addition to HBV, gender (male), smoking, and drinking habits also increase the risk of HCC (5–7). Studies report that the cumulative incidence of HCC in male chronic hepatitis B patients is three times higher than in females (8). HCC secondary to alcohol and chronic hepatitis B infection is more common in men (9). Furthermore, toxic agents in cigarettes and alcohol can exacerbate liver damage and hepatitis, resulting in a higher risk of HCC (7). While numerous studies have explored the risk factors and general outcomes of HCC, limited research has focused specifically on the recurrence and prognosis of male HCC patients with chronic HBV infection who also have long-term smoking and drinking habits, particularly following local ablation treatment. The interplay of these risk factors and their impact on long-term survival remains understudied. Furthermore, despite advances in treatment, there is a lack of predictive models that incorporate both clinical and behavioral factors to guide individualized treatment strategies for this high-risk population.

Therefore, our study addresses this gap by retrospectively analyzing the prognosis of early-stage male HCC patients with chronic HBV infection, combined with smoking and drinking habits, following local ablation therapy. Additionally, we develop a machine learning-based prediction model to provide new insights into personalized treatment and prognosis for this specific group of HCC patients.

## Materials and methods

### Study patients

Data were retrospectively collected from patients treated at Capital Medical University, Beijing Youan Hospital, from 2014 to 2022. The diagnosis of hepatocellular carcinoma (HCC) was based on the American Association for the Study of Liver Diseases guidelines. All patients underwent a single session of radiofrequency ablation (RFA) therapy. Following the inclusion and exclusion criteria, data from 257 patients were collected. Inclusion Criteria were:1. Male; 2. Age 18-75 years old; 3. Chronic HBV infection (hepatitis B surface antigen [HBsAg] and/or HBV DNA positive for over 6 months); 4. BCLC stage 0 or A; 5. Single session of RFA treatment; 6. Chronic history of smoking or alcohol consumption; 7. Complete follow-up data. And the exclusion criteria were:1. Concurrent chronic virus infection; 2. Liver metastases; 3. Concurrent tumors in other organs; 4. Severe organic diseases or severe dysfunction in the heart, lungs, or kidneys; 5. Additional treatments after RFA therapy or multiple RFA sessions.

According to Dietary Guidelines for Chinese residents, male patients whose alcohol consumption exceeded 25g per day were considered having drinking habits (10). Chronic smoking was referred to as a history of smoking for an smoking period of over 4 days per week (11).

### Data collection

Patient data collected included age, complete blood count (CBC), laboratory test results for liver and kidney function, coagulation function, and indicators for HCC evaluation at baseline. CBC included white blood cells (WBC), neutrophils (Neu), lymphocytes (Lym), monocytes (Mon), red blood cells (RBC), hemoglobin (Hb), and platelets (PLT). Liver and kidney function tests included albumin (Alb), aspartate aminotransferase (AST), alanine aminotransferase (ALT), AST/ALT ratio, total bilirubin (TBIL), globulin, bile acid (BA), gamma-glutamyl transferase (GGT), blood urea nitrogen (BUN), uric acid (UA), cholesterol (CHOL), triglycerides (TG), high-density lipoprotein cholesterol (HDL), and low-density lipoprotein cholesterol (LDL). Coagulation function included prothrombin time (PT), prothrombin activity (PTA), international normalized ratio (INR), thrombin time (TT), and fibrinogen (Fib). Indicators for HCC evaluation included alpha-fetoprotein (AFP), tumor size, and tumor number. Additional collected data included smoking and drinking history, and medical history such as hypertension, diabetes, antiviral therapy, alcoholic liver disease, and cirrhosis. Data were anonymized for subsequent analysis.

### Local ablation treatment

All patients underwent a single session of RFA therapy, which was performed subcutaneously and ultrasound-guided. The RFA system used was a Cool-tip RFA system (Tyco, USA). Once the position of the thermal ablation electrode needle reached the target lesion, radiofrequency ablation was initiated. The power setting for each ablation site was generally set to 100-120 W, with an ablation time of 10-15 minutes. The ablation zone completely covered the lesion, with a margin of 0.5 to 1 cm. Treatments were carried out by physicians with over 5 years of experience in interventional therapy.

### Baseline, endpoint, and follow-up

Baseline was determined as the time of HCC diagnosis at the initial visit to our hospital. The endpoint was overall survival (OS), defined as the time until patient death. The last follow-up was conducted on January 1st, 2024. CBC, liver, kidney, and coagulation function, and tumor status were re-examined 1 month after RFA. Patients were then followed up every three months in the first year and every six months in the second year until the endpoint or the end date of follow-up. Follow-up was conducted via outpatient visits, inpatient stays, or telephone calls.

### Statistical analysis

Independent t-tests were used for continuous normally distributed variables between 2 groups, and non-parametric tests were used for comparison among 2 or 3 groups of non-normally distributed variables. For categorical variable comparison between 3 groups, Pearson Chi-squared tests were used. Kaplan-Meier (KM) curves and log-rank tests were used to describe and compare survival rates.

For machine learning, we utilized least absolute shrinkage and selection operator (Lasso) regression and random survival forest (RSF). Lasso regression is a type of linear regression that includes a regularization parameter λ to avoid model overfitting, suitable for high-dimensional data. RSF analyzes data by constructing multiple survival trees using bootstrapping. Variables selected by both machine learning methods were intersected for further analysis.

The model created by machine learning was evaluated in both the training and validation cohorts for discrimination, calibration, and clinical applicability using C index, receiver operating characteristic (ROC) curves, calibration curves, and decision curve analysis (DCA), respectively. Moreover, a nomogram was established to visualize the model, which is a graphical representation used to predict the clinical outcome based on screened variables. It typically consists of multiple scales aligned in parallel layout, where each scale corresponds to a particular variable. By drawing lines between these scales, one can determine the predicted outcome. All analyses were performed using R (version 4.3.2), with p < 0.05 considered statistically significant. (R scripts are available in https://github.com/Sakuflo/male_HBV_HCC_drinking-smoking).

## Results

### Comparison of HCC patients under different risks

To begin with, to determine the prognosis of male patients with smoking and drinking habits, we collected clinical data from 3 groups of patients: patients without these habits (n=298), those with either smoking or drinking habits under similar conditions (n=283) and those with both smoking and drinking habits (n=257). The comparison of clinical data among the groups is presented in **Table 1**. Statistically significant differences were observed between the three groups in RBC, ALT, TBIL, GGT, ALP, bile acid, diabetes, ALD, and cirrhosis.

**Table 1:** Comparison of baseline data of three groups of patients.

	Group 1 N=298	Group 2 N=283	Group 3 N=257	p
Age (mean [SD])	55.54 (10.58)	55.82 (8.67)	56.49 (7.33)	0.452^1^
WBC (median [IQR])	4.90 [3.68, 6.19]	5.03 [3.99, 6.73]	4.90 [3.73, 6.30]	0.308^2^
Neu (median [IQR])	2.85 [2.09, 4.12]	3.05 [2.22, 4.29]	2.78 [2.01, 4.09]	0.063
Lym (median [IQR])	1.20 [0.85, 1.61]	1.21 [0.87, 1.67]	1.23 [0.84, 1.69]	0.471
Mon (median [IQR])	0.38 [0.27, 0.53]	0.40 [0.29, 0.57]	0.38 [0.29, 0.53]	0.330
Eosinophil (median [IQR])	0.11 [0.05, 0.18]	0.10 [0.05, 0.17]	0.12 [0.06, 0.19]	0.250
Basophilia (median [IQR])	0.01 [0.01, 0.02]	0.01 [0.01, 0.02]	0.01 [0.01, 0.02]	0.848
RBC (median [IQR])	4.38 [3.96, 4.74]	4.28 [3.87, 4.65]	4.28 [3.78, 4.60]	0.037
Hb (median [IQR])	138.00 [124.00, 148.00]	135.00 [122.25, 147.00]	136.00 [121.00, 148.00]	0.452
PLT (median [IQR])	114.00 [77.00, 160.75]	116.50 [73.25, 159.00]	116.00 [76.00, 159.00]	0.996
ALT (median [IQR])	28.35 [20.20, 38.88]	30.00 [21.12, 39.25]	26.10 [19.00, 35.70]	0.021
AST (median [IQR])	28.00 [21.85, 35.60]	28.20 [23.52, 36.48]	28.70 [22.00, 37.00]	0.421
AST/ALT (median [IQR])	1.02 [0.78, 1.26]	1.00 [0.77, 1.29]	1.05 [0.84, 1.40]	0.094
TBIL (median [IQR])	18.45 [13.40, 24.28]	16.30 [11.62, 23.17]	16.40 [11.70, 22.00]	0.025
Alb (median [IQR])	38.10 [34.90, 40.60]	37.35 [34.30, 40.53]	37.50 [34.40, 40.30]	0.476
Globulin (median [IQR])	27.90 [24.72, 31.37]	27.25 [24.02, 31.37]	27.50 [24.10, 31.40]	0.505
GGT (median [IQR])	46.80 [29.08, 75.80]	57.00 [38.00, 92.20]	62.00 [35.00, 103.00]	<0.001
ALP (median [IQR])	77.10 [61.62, 97.45]	80.80 [66.58, 107.97]	82.00 [66.40, 103.20]	0.027
PALB (median [IQR])	141.00 [97.50, 186.50]	132.00 [90.10, 180.00]	138.00 [101.60, 176.45]	0.250
Bile acid (median [IQR])	9.95 [5.10, 22.00]	10.45 [5.82, 24.50]	13.20 [6.70, 26.40]	0.046
Cr (median [IQR])	67.10 [58.00, 76.97]	64.45 [57.73, 74.75]	65.00 [58.00, 73.75]	0.191
Uric acid (median [IQR])	285.00 [234.10, 346.75]	275.50 [231.32, 333.92]	287.40 [241.05, 352.17]	0.116
CHOL(median [IQR])	3.70 [3.21, 4.34]	3.70 [3.24, 4.30]	3.80 [3.34, 4.34]	0.378
TG (median [IQR])	0.96 [0.78, 1.23]	0.97 [0.77, 1.30]	0.93 [0.73, 1.28]	0.695
HDL (median [IQR])	1.07 [0.90, 1.26]	1.02 [0.85, 1.28]	1.06 [0.86, 1.31]	0.650
LDL (median [IQR])	2.24 [1.68, 2.64]	2.08 [1.69, 2.63]	2.15 [1.67, 2.69]	0.766
PT (median [IQR])	12.40 [11.70, 13.30]	12.35 [11.50, 13.50]	12.20 [11.47, 13.30]	0.188
PTA (median [IQR])	85.00 [77.00, 93.00]	86.00 [75.25, 95.00]	86.00 [78.00, 96.70]	0.226
INR (median [IQR])	1.10 [1.04, 1.18]	1.10 [1.02, 1.19]	1.09 [1.02, 1.17]	0.172
Fib (median [IQR])	2.56 [2.16, 3.08]	2.65 [2.19, 3.36]	2.76 [2.25, 3.36]	0.077
TT (median [IQR])	15.70 [14.30, 17.10]	15.60 [14.10, 17.30]	15.50 [14.10, 17.30]	0.814
AFP (mean (SD))	309.75 (2040.27)	419.68 (2045.96)	226.90 (790.84)	0.444
Tumor size (mean [SD])	26.03(14.26)	27.99(16.76)	26.22(16.60)	0.270
Tumor number (mean [SD])	1.46(0.88)	1.50(0.84)	1.53(0.87)	0.666
Hypertension No Yes	227 71	208 75	186 71	0.571^3^
Diabetes No Yes	248 50	227 56	183 74	0.002
Antiviral therapy No Yes	136 162	122 161	122 135	0.592
ALD No Yes	293 5	229 54	99 158	<0.001
Cirrhosis No Yes	30 268	54 229	27 230	0.002

Group 1: patients without smoking or drinking habits; Group 2: patients with either smoking or drinking habit; Group 3: patients with smoking and drinking habits. Abbreviations and units: WBC, White blood cells(×10^9); Neu, neutrophils(×10^9); Lym, lymphocytes(×10^9); Mon, monocytes(×10^9); RBC, red blood cells(×10^9); Hb, hemoglobin(g/L); PLT, platelets(×10^9); Alb, albumin(g/L); AST, aspartate aminotransferase(U/L); ALT, alanine aminotransferase(U/L); TBIL, total bilirubin(μmol/L); PALB, pre-albumin(mg/L); GGT, gamma-glutamyl transferase(U/L); ALP, alkaline phosphate(U/L); CHOL, cholesterol(mmol/L); TG, triglycerides(mmol/L); HDL, high-density lipoprotein cholesterol(mmol/L); LDL, low-density lipoprotein cholesterol(mmol/L); Cr, Creatine(μmol/L); PT, prothrombin time(s); PTA, prothrombin activity(%); INR, international normalized ratio; TT, thrombin time(s); Fib, fibrinogen(g/L); AFP, alpha-fetoprotein(ng/mL); Tumor size(mm); ALD, alcoholic liver disease.1: independent t test; 2: non-parametric test; 3: Pearson Chi-squared test.

**Figure 1:**
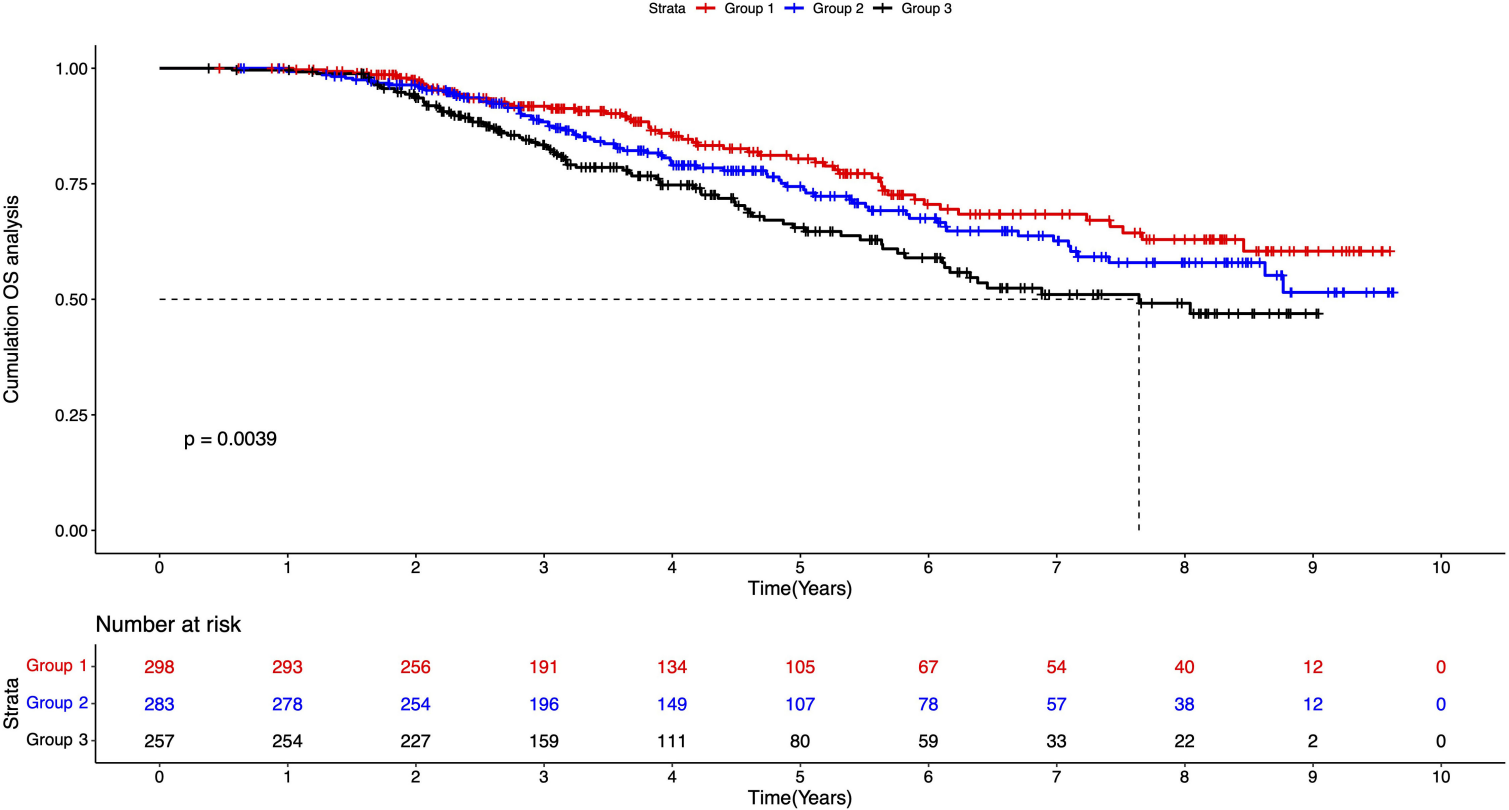
K-M survival plot of three groups of patients. Group 1: patients without smoking or drinking habits; Group 2: patients with either smoking or drinking habit; Group 3: patients with smoking and drinking habits.

We further plotted the Kaplan-Meier (K-M) curves to calculate and compare the survival rates of the three groups (**Figure 1**). Until the end of the follow-up date, the complete median overall survival (mOS) was not available for groups 1 and 2; while in group 3, the mOS was 7.651 years, which suggested that Group 3 had worse survival performance. The log-rank test showed that the cumulative OS was statistically significant between the three groups. The survival rate in group 3 decreased significantly, indicating a poorer prognosis for patients with both smoking and drinking habits.

### Screening risk factors by Lasso and multi-variate Cox regression

Based on the poorer prognosis of patients with smoking and drinking habits, we divided the 257 patients to training and validation set according to 7:3, using machine learning methods and multi-variate Cox regression to screen the risk factors, finally gaining the variables that affect survival. There were no significant differences among training and validation set, so the model we established in training set was able to fit the validation set.(**Table 2**).

**Table 2:** Comparison of clinical data between training and validation cohort.

	Training cohort(N=179)	Validation Cohort (N=78)	P
Age (mean [SD])	55.54(10.58)	55.82(8.67)	0.358^1^
WBC (median[IQR])	4.93 [3.67, 6.36]	4.80 [3.78, 6.23]	0.846^2^
Neu (median[IQR])	2.75 [2.00, 4.14]	2.92 [2.03, 3.93]	0.780
Lym (median[IQR])	1.22 [0.86, 1.69]	1.26 [0.86, 1.66]	0.874
Mon (median[IQR])	0.38 [0.28, 0.52]	0.36 [0.29, 0.53]	0.867
Eosinophil (median[IQR])	0.12 [0.05, 0.19]	0.12 [0.06, 0.20]	0.832
Basophil (median[IQR])	0.01 [0.01, 0.02]	0.01 [0.01, 0.02]	0.974
RBC (median[IQR])	4.25 [3.74, 4.56]	4.42 [3.86, 4.74]	0.061
Hb (median[IQR])	135.00 [119.00, 146.50]	139.50 [124.25, 149.00]	0.111
PLT (median[IQR])	113.00 [76.50, 160.50]	128.50 [75.00, 157.25]	0.964
ALT (median[IQR])	26.50 [19.25, 37.30]	26.00 [19.00, 34.28]	0.512
AST (median[IQR])	28.70 [22.40, 37.00]	28.00 [21.68, 34.80]	0.251
AST/ALT (median[IQR])	1.04 [0.84, 1.40]	1.06 [0.85, 1.35]	0.934
TBIL (median[IQR])	16.60 [11.60, 23.55]	15.90 [11.83, 21.35]	0.459
Alb (median[IQR])	37.50 [34.40, 40.10]	37.65 [34.82, 40.48]	0.675
Globulin (median[IQR])	27.40 [24.30, 30.70]	27.65 [24.02, 31.60]	0.691
GGT (median[IQR])	58.40 [33.10, 101.00]	65.15 [40.55, 107.67]	0.435
ALP (median[IQR])	82.10 [69.00, 104.70]	81.35 [63.17, 101.92]	0.435
PALB (median[IQR])	135.85 [96.85, 177.23]	144.50 [109.40, 174.50]	0.471
Bile acid (median[IQR])	13.10 [5.95, 25.20]	14.30 [7.82, 26.48]	0.205
Cr (median[IQR])	65.80 [58.55, 73.92]	64.20 [58.00, 72.75]	0.622
CHOL (median[IQR])	3.82 [3.32, 4.47]	3.79 [3.36, 4.23]	0.530
TG (median[IQR])	0.94 [0.73, 1.24]	0.92 [0.74, 1.33]	0.830
HDL (median[IQR])	1.06 [0.83, 1.31]	1.08 [0.89, 1.28]	0.689
LDL (median[IQR])	2.03 [1.64, 2.58]	2.24 [1.92, 2.86]	0.060
PT (median[IQR])	12.30 [11.50, 13.20]	12.15 [11.25, 13.38]	0.854
PTA (median[IQR])	85.80 [78.17, 95.45]	88.50 [76.25, 98.75]	0.558
INR (median[IQR])	1.10 [1.02, 1.15]	1.08 [1.00, 1.19]	0.792
TT (median[IQR])	15.70 [14.30, 17.45]	15.25 [13.62, 16.65]	0.562
Fib (median[IQR])	2.74 [2.23, 3.37]	2.83 [2.33, 3.36]	0.562
AFP (mean[SD])	207.81 (739.43)	269.98 (899.80)	0.952
Tumor size(mean [SD])	26.07(16.69)	26.55(16.48)	0.832
Tumor number(mean [SD])	1.58(0.92)	1.41(0.71)	0.160
Anti-viral therapy			
No	85	37	0.994^3^
Yes	94	41	
ALD			
No	73	26	0.259
Yes	106	52	
Diabetes			
No	131	52	0.289
Yes	48	26	
Hypertension			
No	127	59	0.439
Yes	52	19	
Cirrhosis			
No	22	5	0.158
Yes	157	73	

Abbreviations and units: WBC, White blood cells(×10^9); Neu, neutrophils(×10^9); Lym, lymphocytes(×10^9); Mon, monocytes(×10^9); RBC, red blood cells(×10^9); Hb, hemoglobin(g/L); PLT, platelets(×10^9); Alb, albumin(g/L); AST, aspartate aminotransferase(U/L); ALT, alanine aminotransferase(U/L); TBIL, total bilirubin(μmol/L); PALB, pre-albumin(mg/L); bile acid(μmol/L); GGT, gamma-glutamyl transferase(U/L); ALP, alkaline phosphate(U/L); CHOL, cholesterol(mmol/L); TG, triglycerides(mmol/L); HDL, high-density lipoprotein cholesterol(mmol/L); LDL, low-density lipoprotein cholesterol(mmol/L); Cr, Creatine(μmol/L); PT, prothrombin time(s); PTA, prothrombin activity(%); INR, international normalized ratio; TT, thrombin time(s); Fib, fibrinogen(g/L); AFP, alpha-fetoprotein(ng/mL); Tumor size(mm); ALD, alcoholic liver disease.1: independent t test; 2: non-parametric test; 3: Pearson Chi-square test.

**Figure 2:**
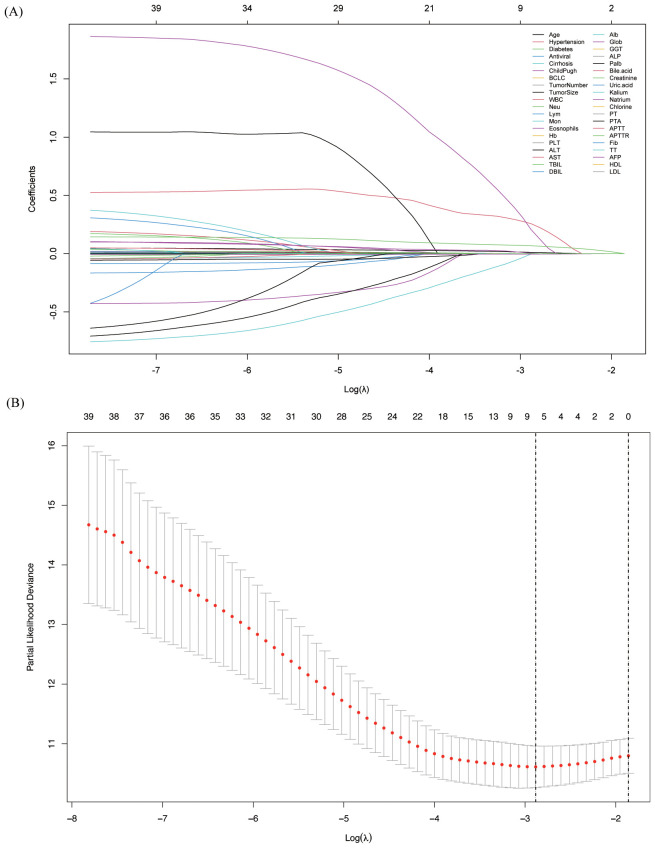
Variable screening using Lasso regression and 10-fold cross-validation. **(A)** Lasso coefficient path of different variables, showing the shrinkage effect as the regularization parameter changes; **(B)** Selection of the optimal lambda value using 10-fold cross-validation to identify key predictors.

In the training cohort, we initially applied Lasso regression and plotted the Lasso path to illustrate how the coefficients change with increasing λ (**Figure 2A**). We then used 10-fold cross-validation to calculate the partial likelihood deviance for different λ values (**Figure 2B**). When λ = 0.0559, the partial likelihood deviance was the smallest, indicating that this was the best-fitting model. At this λ value, there were 8 variables with non-zero coefficients, which were subsequently included in the model: age, tumor number, lymphocyte, monocyte, globulin, ALP, PALB, and TT.

**Figure 3:**
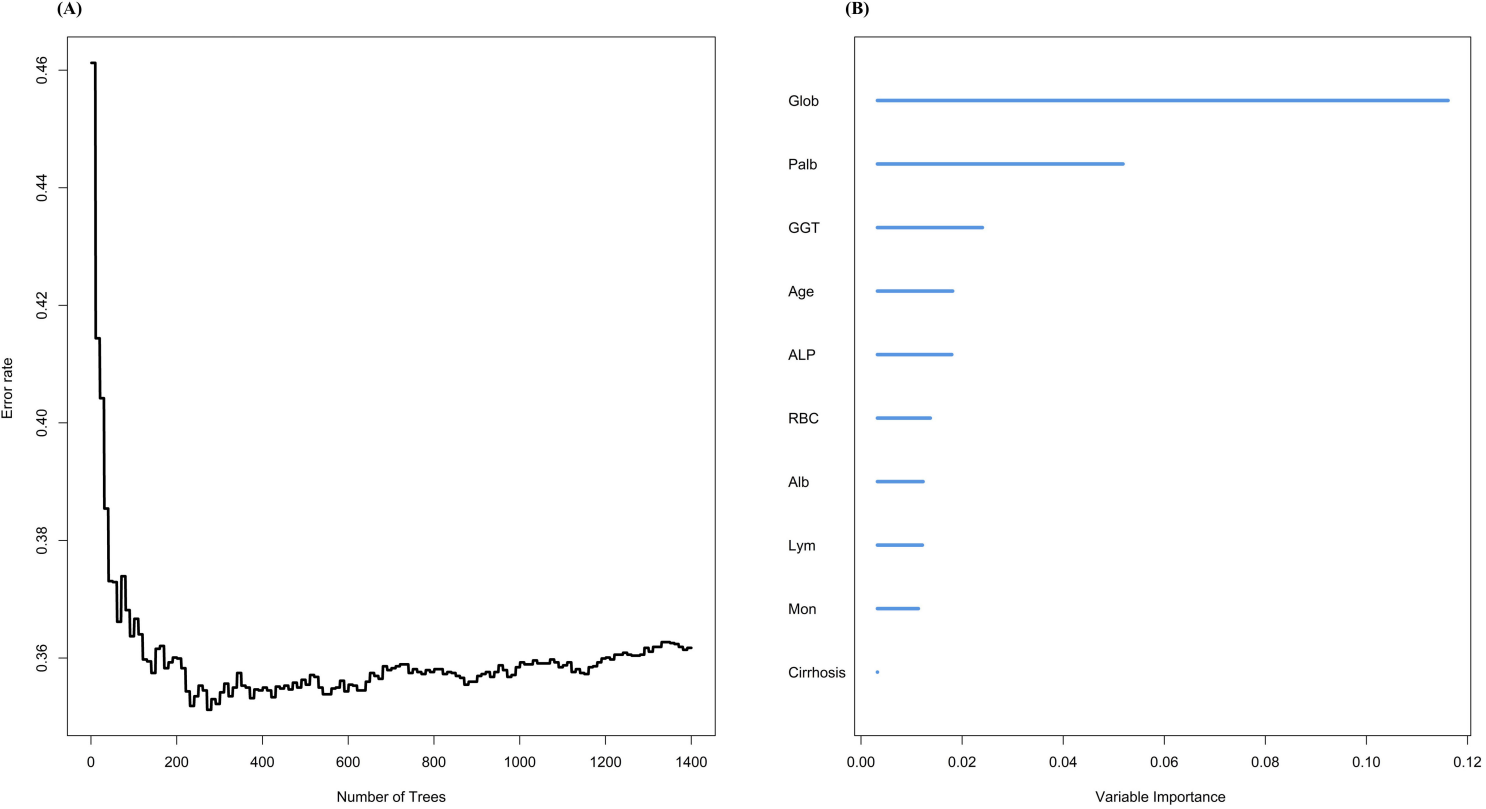
Variable screening using random survival forest. **(A)**The relationship between different number of trees and error rate; **(B)**VIMP of different variables under RSF model.

Next, we used another machine learning method, RSF, to screen the variables (**Figure 3**). First, we constructed decision trees ranging from 0 to 1400, and it is clear that the error rate reaches its lowest point and stabilizes after 200 trees. At this stage, the model demonstrates strong predictive ability. Based on this optimized model, we calculated the variable importance measures (VIMP). The top 10 variables ranked by VIMP were globulin, PALB, GGT, age, ALP, RBC, Alb, lymphocyte, monocyte, and cirrhosis.

Next, we intersected the variables screened by the two machine learning methods and identified age, globulin, PALB, ALP, lymphocyte, and monocyte. These variables were then subjected to multivariate Cox regression analysis, which revealed that monocyte, globulin, and PALB had the most significant influence on OS (p<0.05). Monocyte and globulin were identified as risk factors (HR>1), while PALB was found to be a protective factor (HR<1) [**Table 3**].

**Table 3:** Multivariate Cox regression of hazard ratio.

	HR (95%CI)	P
Age	1.031 (0.992-1.072)	0.118
Lym	0.654 (0.428-1.001)	0.051
**Mon**	**3.664 (1.473-9.113)**	**0.005**
**Globulin**	**1.11 7(1.057-1.18)**	**<0.001**
ALP	1.002 (0.997-1.007)	0.476
**PALB**	**0.994 (0.989-0.999)**	**0.028**

Lym, lymphocytes; Mon, monocytes; ALP, alkaline phosphatase; PALB, pre-albumin.

Mon, Globulin, PALB and the results are bold to indicate variables selected.

### Establishment of Nomogram

**Figure 4:**
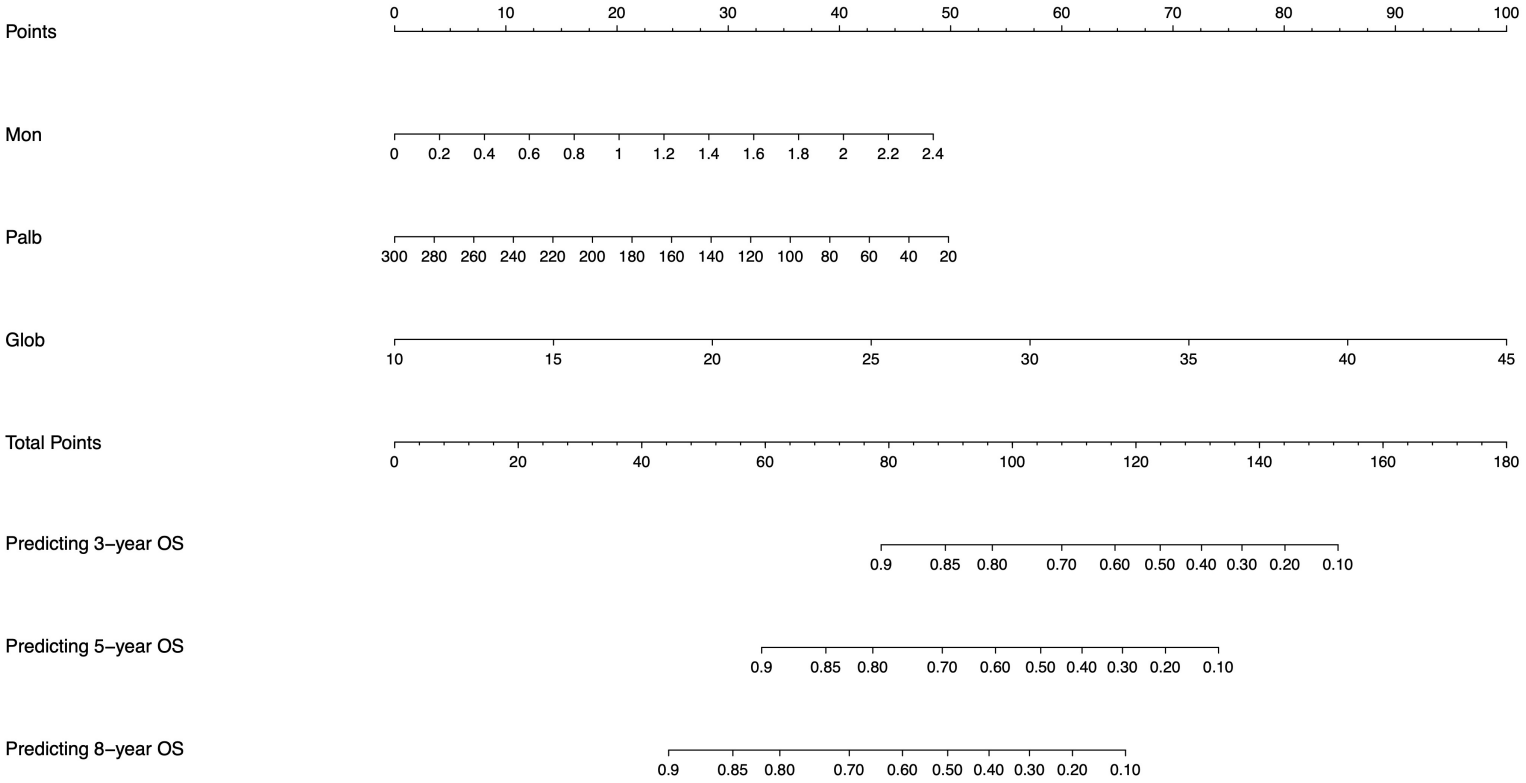
Nomogram generated from the machine learning model for individualized risk prediction in patients, integrating significant risk factors identified in the study.

We converted our mathematical model calculated by machine learning to a visualized nomogram to evaluate and categorize patients specifically (**Figure 4**). Based on the specific values of monocytes, PALB and globulin of the patient, the scores corresponding to Points can be found vertically upwards, the scores of each score are aggregated, and the corresponding values are found in total Points and vertically downwards, and the probability of their predicting 3-year, 5-year and 8-year OS survival can be found.

### Evaluation for discrimination, accuracy and clinical applicability

**Figure 5:**
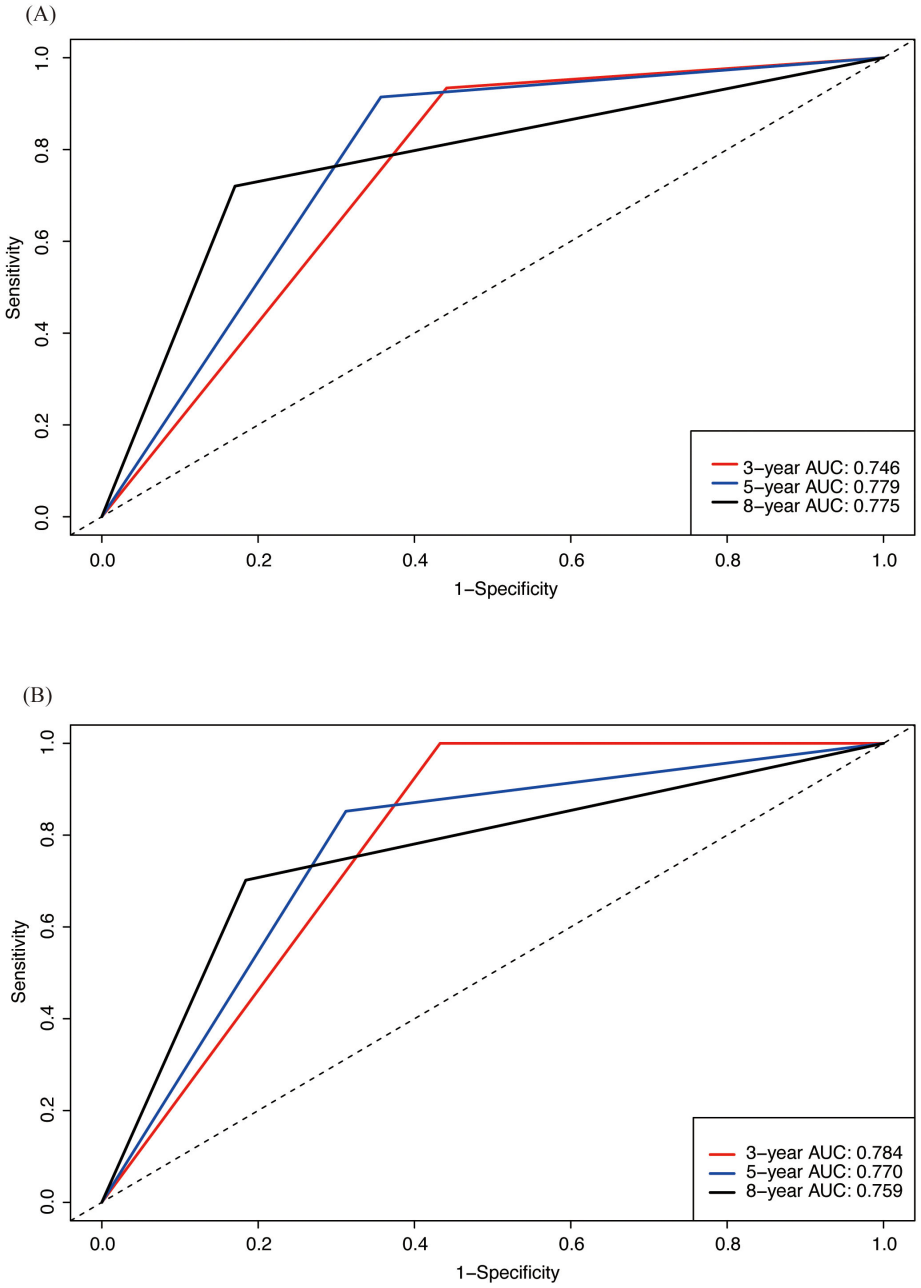
ROC analysis assessing the predictive performance of nomogram for OS in both training and validation cohorts. **(A)** ROC analysis in training cohort; **(B)** ROC analysis in validation cohort.

Firstly, we used C-index and ROC analysis to evaluate the ability of Nomogram to discriminate between patients in the training set and validation set at 3, 5 and 8 years (**Figure 5**). The C-index was 0.712 (95% CI 0.663-0.761) in the training cohort and 0.719 (95% CI 0.648-0.789) in the validation cohort, which reflected good predicting ability. ROC analysis showed that in the training set, the area under curve (AUC) of OS at 3, 5, and 8 years are 0.746,0.779, and 0.775, respectively. In the validation set, it is 0.784,0.770, and 0.759, respectively, which showed that the model has a good discriminatory ability in both the training and validation sets.

**Figure 6:**
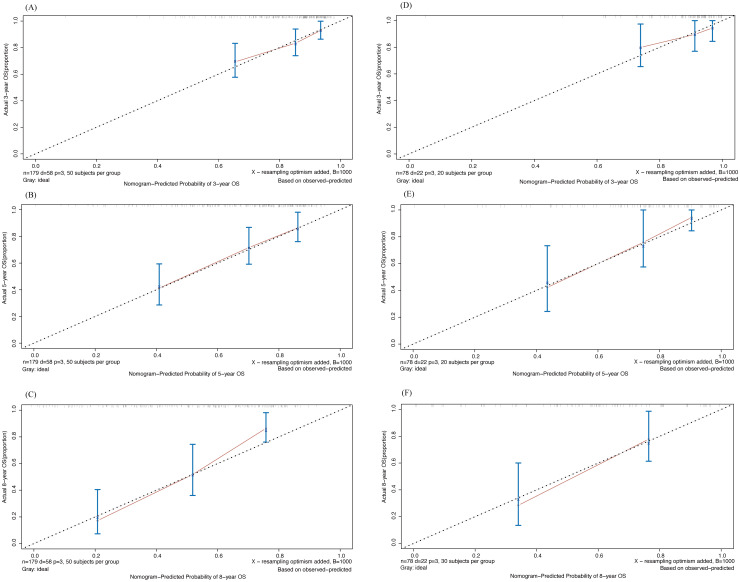
Calibration curve of 3-,5- and 8-year OS drawn by Nomogram in training and validation cohort. **(A-C)** 3-, 5- and 8-year OS in training cohort; **(D-F)** 3-, 5- and 8-year OS in validation cohort.

Next, we assessed the accuracy of the model using the calibration curve (**Figure 6**). The horizontal coordinate was the predicted survival probability and the vertical coordinate was the actual observed survival rate. The diagonal dashed line represented the ideal case of complete calibration, and the closer the line between the points was to the diagonal line, the higher the prediction accuracy is represented. It can be observed that Nomogram’s predictions for 3-, 5-, and 8-year were closer to the diagonal line in both the training and validation sets, showing good accuracy.

**Figure 7:**
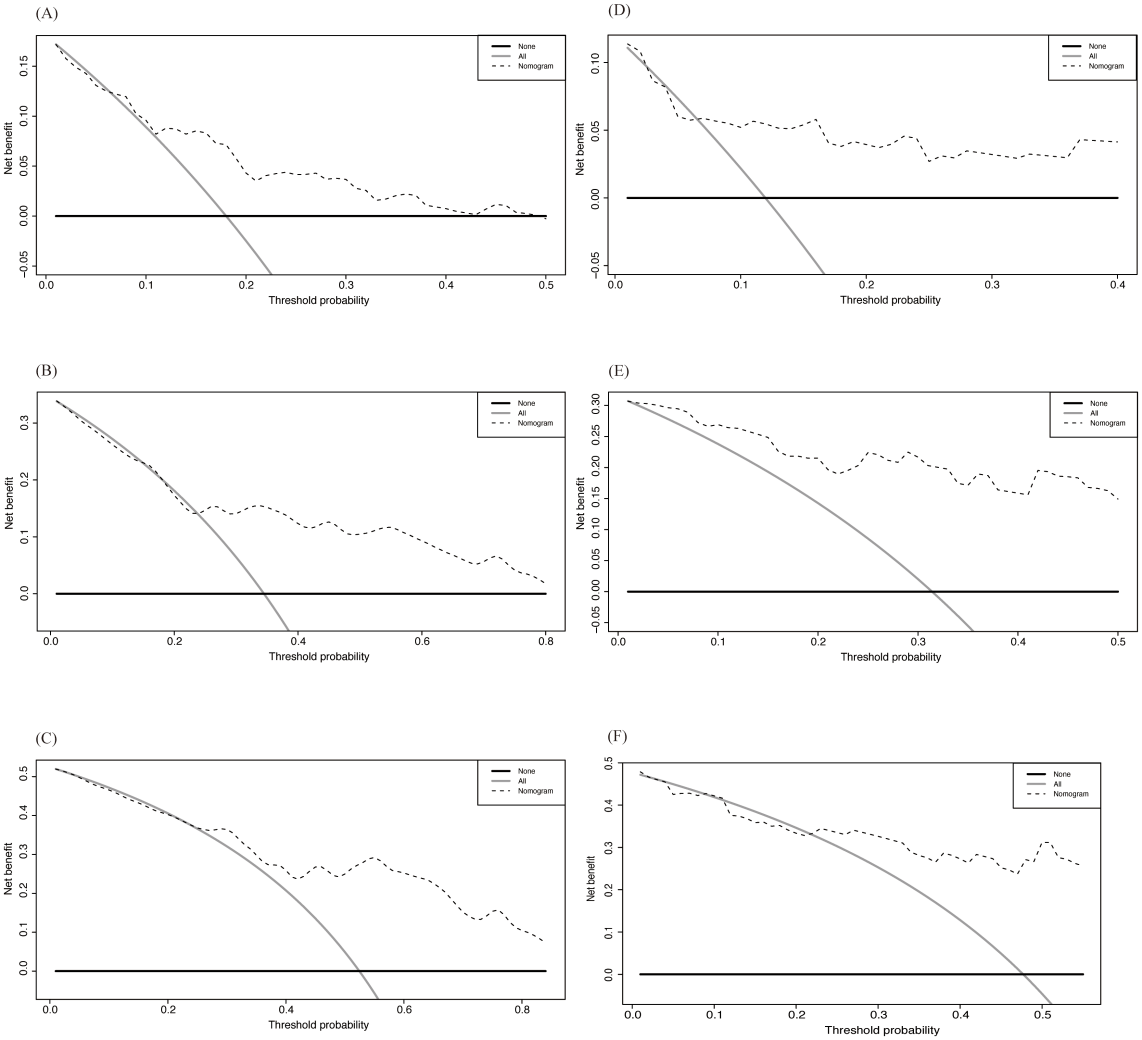
DCA of Nomogram in training and validation cohort. **(A-C)** 3-, 5- and 8-year OS in training cohort; **(D-F)** 3-, 5- and 8-year OS in validation cohort.

We then used Decision Curve Analysis (DCA) to assess the clinical applicability of the nomogram (**Figure 7**). The analysis evaluated the prediction results and the effect of intervention by calculating the net benefit across different threshold probabilities (0 to 1). The horizontal axis represents the threshold probability, while the vertical axis represents the net benefit. The black solid curve represents the net benefit with all interventions, the black horizontal solid line represents the net benefit without any interventions, and the dashed line represents the net benefit when using the nomogram for prediction. As shown in the figure, despite some differences between the training and validation sets, the net benefit curve of the nomogram was higher than that of the all-intervention approach for most of the threshold range, indicating overall good performance.

### K-M survival curve assessment of patients using Nomogram

**Figure 8:**
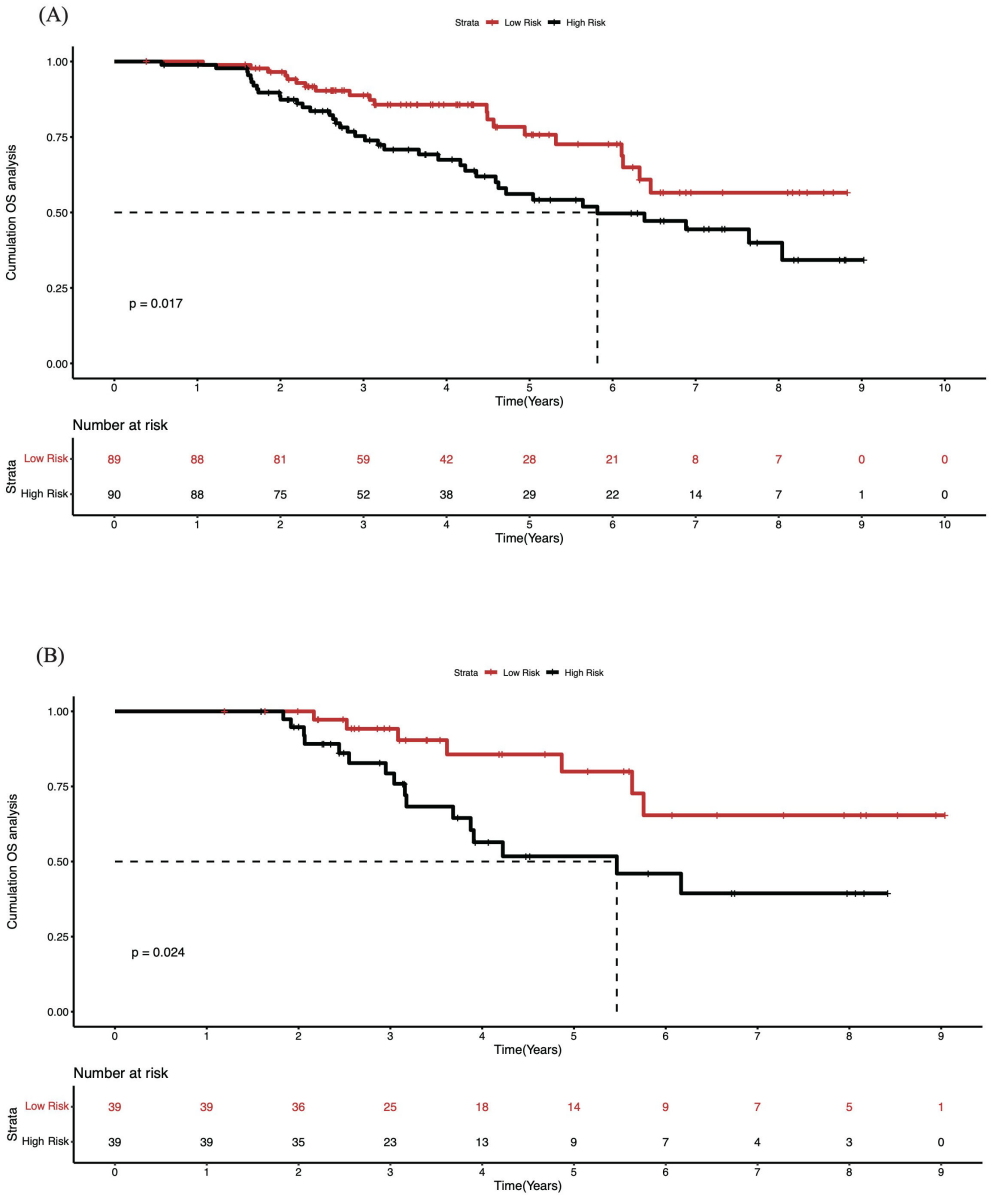
K-M survival curve stratifying patients into high- and low-risk groups based on nomogram in training and validation cohort. **(A)** Survival curve in the training cohort; **(B)** Survival curve in the validation cohort, showing distinct survival differences between risk groups.

Based on the Nomogram score, we classified patients into high-risk group (top 50%) and low-risk group (bottom 50%) according to from high to low (**Figure 8**). The mOS of the high-risk group in the training set was 5.46 years, and the mOS of the low-risk group was not obtained up to the endpoint, and the results in the validation group were similar to those in the training set (5.81 years for the high mOS and no mOS for the low-risk group). In the training set, the OS of patients in the low-risk group was 0.94, 0.80, and 0.65 at 3, 5, and 8 years, respectively; in the high-risk group, it was 0.79, 0.52, and 0.39, respectively. All of the above results showed that the prognosis of the low-risk group was better than that of the high-risk group, suggesting that the Nomogram established based on the results of the machine learning has a good ability of prognostic assessment and application value.

## Discussion

Local ablation therapy is one of the recommended treatments for patients with BCLC stage 0 and A HCC. However, due to its high recurrence rate, more research is needed to explore the prognosis after ablation therapy, particularly for high-risk patients. Public health research has shown that has shown that men surpass women in both the typical frequency and amount of smoking and drinking based on various social economical or cultural reasons, as well as in the incidence of adverse consequences related to smoking and drinking, which both are risks of high recurrence rate of HCC (7, 12, 13). In this study, we evaluated the prognosis of male patients with chronic HBV infection and smoking and drinking habits after HCC ablation therapy. We first confirmed that the 3-, 5-, and 8-year OS in this group were worse. We then used machine learning methods to propose a new scoring system for this group, providing new strategies for follow-up and intervention timing.

Chronic HBV infection is a known high-risk factor for HCC. The inflammatory environment caused by the infection and the integration of viral DNA into the host are key reasons for carcinogenesis, which will not be elaborated here (14). Among gender factors, the incidence and recurrence rates of HCC in men are 2-3 times higher than in women (15). It is generally accepted that the differences in sex hormone levels due to gender are an important reason for the different risks of disease between men and women. In HCC, androgens and androgen receptors promote cell proliferation, migration, and invasion, while inhibiting apoptosis (16). Additionally, the risk factors for HCC are not balanced between men and women. Men have higher rates of smoking and heavy drinking than women (15). Cigarette smoke contains various compounds harmful to the liver. For example, 4-aminobiphenyl in cigarette smoke forms 4-aminobiphenyl-DNA adducts in liver cells. When stratified by the levels of these adducts, the risk of HCC in the high-level group is 10 times higher than in the low-level group. When combined with HBV infection, the risk is 40 times higher in patients with positive HBsAg and high adduct levels compared to those with negative HBsAg and low adduct levels (17). Other carcinogenic chemicals, such as nitrosamines, can induce a systemic inflammatory state, elevate various cytokine levels, and promote DNA mutations and cell proliferation (18). Long-term heavy drinking not only increases the risk of alcoholic liver disease but is also a direct risk factor for carcinogenesis. The metabolism of ethanol exacerbates mitochondrial oxidative stress, and the intermediate metabolite acetaldehyde stimulates the increase of reactive oxygen species (ROS), causing mitochondrial damage and abnormal lipid metabolism in liver cells, making them more prone to necrosis (19). In summary, our grouping of subjects considered multiple high-risk factors for HCC, which has important clinical significance.

In selecting variables, we utilized two machine learning methods: Lasso regression and Random Survival Forest (RSF), followed by multivariate Cox regression, and ultimately identified monocytes, globulin, and pre-albumin. Using both machine learning techniques enhances model robustness, reduces the risk of overfitting, and mitigates biases introduced by a single method. Lasso feature selection is based on linear relationships and L1 regularization, effectively addressing multicollinearity issues, while RSF evaluates variable importance through a nonlinear decision tree model. Taking the intersection of variables selected by both methods ensures their importance under both linear and nonlinear relationships, thereby improving model interpretability.

Based on this, the nomogram we established demonstrated superior performance, effectively distinguishing between high-risk and low-risk patients in terms of 3-, 5-, and 8-year overall survival (OS). In this study, monocytes were identified as a risk factor for recurrence in the study population (HR=3.664, 95% CI 1.473-9.113). Similar findings were reported by Hong et al. In studies related to prognostic models for HCC, monocytes are often included in the form of the monocyte-to-lymphocyte ratio (MLR) (20). Numerous studies across different populations have confirmed that a high MLR is a risk factor for HCC recurrence. In a clinical predictive model for AFP-negative HCC patients, MLR was identified as a risk factor for recurrence and was included as one of the variables in the predictive model (21). Wang et al. found that a high MLR was significantly associated with recurrence and decreased overall survival in patients undergoing TACE combined with ablation therapy (22).

A high number of peripheral blood monocytes suggests enhanced patrolling under tumor conditions, which can migrate to liver tumor tissues and differentiate into tumor-associated macrophages (TAMs). These TAMs secrete cytokines such as TGF-beta and IL-10, promoting tumor growth and suppressing tumor immunity, thus forming part of the tumor microenvironment (23). Elevated globulin levels typically indicate chronic liver disease, and pre-albumin (PALB) can reflect hepatocyte damage earlier than albumin (Alb). A decrease in PALB indicates systemic inflammation and malignancy, which aligns with our findings that globulin is a risk factor, while PALB is a protective factor.

There are several limitations in our study. Firstly, we did not stratify patients based on the degree of smoking and drinking. According to some studies, the risks vary with different levels of smoking and drinking. Secondly, our data was collected from a single center with a limited patient cohort, which may introduce a potential limitation in the generalizability of our findings. The limited sample size may increase the risk of overfitting in our model, meaning that while the model performs well on the current dataset, its predictive power may be less reliable when applied to external datasets. Thus, further external validations with larger and more diverse cohorts are necessary in the future to improve the robustness and generalizability of our predictive model.

Despite all the limitations, our research still provided a promising prediction model, which serves as a tool for clinical decision-making, enabling precise monitoring and intervention for high-risk populations. This approach not only helps improve patient survival rates but also lays the foundation for future multi-center validation studies and long-term follow-up research. Specifically, we suggest that these patients through the nomogram should be monitored more frequently and considered for adjunct therapies post-ablation to reduce recurrence risk.

## Conclusion

In this study, we evaluated the OS of high-risk HCC in male patients with chronic HBV infection and smoking and drinking habits. Using Lasso regression, RSF, and Cox regression, we identified four key variables to develop a clinically practical nomogram. This nomogram, validated for its accuracy and clinical applicability, effectively stratified patients and assessed their OS, offering valuable guidance for follow-up and treatment timing.

## Data Availability

The raw data supporting the conclusions of this article will be made available by the authors, without undue reservation.
